# The Changes in Stress Coping, Alcohol Use, Cigarette Smoking and Physical Activity during COVID-19 Related Lockdown in Medical Students in Poland

**DOI:** 10.3390/ijerph19010302

**Published:** 2021-12-28

**Authors:** Aureliusz Kosendiak, Magdalena Król, Milena Ściskalska, Marta Kepinska

**Affiliations:** 1 Department of Physical Education and Sport, Wroclaw Medical University, Wojciecha z Brudzewa 12, 51-601 Wroclaw, Poland; aureliusz.kosendiak@umw.edu.pl; 2 Department of Pharmaceutical Biochemistry, Division of Biomedical and Environmental Analyses, Faculty of Pharmacy, Wroclaw Medical University, Borowska 211a, 50-556 Wroclaw, Poland; mag.krol@student.umw.edu.pl (M.K.); milena.sciskalska@umw.edu.pl (M.Ś.)

**Keywords:** alcohol, COVID-19, distress, physical activity, smoking

## Abstract

The ongoing COVID-19 pandemic has significantly limited social contacts, thus contributing to deepening isolation. Therefore, SARS-CoV-2 exerted on humanity not only a physical impact but also a psychological one, often increasing the feeling of stress. The long-term effects of such a state could include the management of depression, so our study aimed to analyze groups of medical students in different periods of the pandemic (at the beginning of the pandemic, after half a year of the pandemic, after one year of the pandemic) in order to assess the impact of this situation on coping with stress. The impact of the pandemic on the development of stress factors such as alcohol consumption and smoking was also studied. The level of physical activity in the context of coping with an uncertain situation was also assessed. The impact of the above-mentioned factors on the behavior of students, including the Mini-COPE questionnaire, AUDIT test, the Fagerström test and the IPAQ questionnaire was analyzed. It has been shown that as the pandemic and the lockdown progressed, patients consumed more often or larger amounts of alcohol, smoked more cigarettes, and levels of physical activity decreased. All these factors may have had some impact on the deterioration of coping with stress among the respondents, which would indicate that the COVID-19 pandemic significantly contributed to an increase in the sense of stress among the students.

## 1. Introduction

 In December 2019 the attention of the world was drawn to the events taking place in the Chinese city, Wuhan, which were to determine the next several months of life for many people [1]. It was there that the first cases of the pneumonia-related disease were reported [2]. In early 2020 Chinese researchers isolated a new virus called SARS-CoV-2 due to its similarity to the structure of the coronaviruses causing severe acute respiratory syndrome (SARS), and the disease itself began to be referred to as COVID-19 [3]. The trouble-free movement of people between countries in the modern world has contributed to the rapid increase in infections not only in China or even Asia, but also in countries belonging to other continents. In response to such developments, in March 2020 the World Health Organization (WHO) declared a pandemic [4].

 COVID-19 caused social and economic disruptions at a global level, which were reflected in, among others, widespread shortages in supplies caused by mass buying-out of products [5]. It was stipulated that many sports, cultural, religious and political events be canceled [6]. Moreover, the changes in life required by the attempt to contain the pandemic were sudden and implemented almost immediately. The limitation of direct interpersonal contacts has condemned many people to social isolation, which, in combination with quarantine and lockdown, may generate even greater stress [7]. The need to adapt to the new style of learning, working and dealing with everyday matters caused a sense of insecurity, fear and anxiety for their own health, and that of their friends and family. Financial problems due to restrictions imposed on the economy turned out to be an additional stressor, which in turn led to mass layoffs [8]. Another stressor was certainly the rapid spread of the new coronavirus and its high contagiousness, as well as difficulties in accessing healthcare, which also turned out to have a large impact on the behavior of the general public [9,10].

On 12 March 2020, a state of emergency of epidemic was declared in Poland, that was associated with a ban on public, state and religious gatherings of more than 50 people, the restaurants only functioning in the take-out form, closing of gyms, swimming pools, dance clubs, fitness clubs, museums, libraries and cinemas. In March and October 2020, there was a sanitary regime in Poland, where learning (schools and university) was only online, excluding practical classes, and above mentioned changes were introduced once again [11,12]. In March 2021, the number of new cases of COVID-19 and hospitalized people was growing significantly. A decision was made to introduce extended safety rules in Poland, and it was recommended to work remotely wherever possible. Isolation, social distancing, and closure of educational institutions, workplaces and entertainment venues affect social health [11]. It was reported that the above-mentioned social inconveniences could lead to emotional outbursts, irritation and change in sleeping and eating habits [13]. These features of behavior can result in deterioration of mood and frustration [14]. Students are a group particularly exposed to various types of stressors due to the sudden need for distance learning, uncertainty about further education and the unstable labor market. Social isolation may additionally result in a direct increase in the occurrence of depressive symptoms in students or their worsening due to stress [15]. Impellizzeri et al. [16] noted that stress particularly affected women aged 21–45. Perhaps this is because this cross-section shows women of childbearing age who are additionally concerned about their offspring or the possibility of becoming pregnant.

Stress is also a major risk factor for the onset and persistence of alcohol abuse [17]. It is impacting many populations where alcohol consumption has indeed increased during the course of the pandemic [18,19]. The respondents also pointed out that they used alcohol more often when they felt increased anxiety or sadness [19]. It is the same with smoking. On the one hand, the closure of campuses and the return of some students to their family homes were certainly not conducive to this addiction, but the growing feeling of anxiety and stress could cause more frequent smoking [20]. Moreover, in some countries, such as Mexico, the number of people trying to quit smoking has decreased during the COVID-19 pandemic [21].

Another problem that the pandemic has placed on society is exercise. Some people were simply forced to resign from it for health reasons, for example because of hospitalization and convalescence. In turn, others were restrained by imposed warnings, including closing gyms and bans on going out into the fresh air. Interestingly, in Spain especially students and people who had been very active significantly reduced their activity and switched to a sedentary lifestyle [22]. Similarly, in other countries, the studies carried out indicate a decrease in the level of social activity due to the sanitary regime and the need to maintain social distance [23]. This is all the more worrying as exercise can help manage stress caused by the pandemic and lockdown. Physical activity and sports have a positive effect on well-being, even in times when society is not under such extreme conditions as a pandemic. In turn, students are a group that is extremely exposed to stressors. Bland et al. [24] demonstrated a positive, protective effect of behavior related to physical activity on stress tolerance among students. Exercise has also been shown to benefit (both physically and mentally) people with high levels of anxiety [25]. Physical activity is also associated with resistance to occupational stress, especially in healthcare professionals [26]. The impact of a sedentary lifestyle on the development of stress is also analyzed. Lee et al. [27] showed that with increasing sedentary lifestyle, the levels of stress, anxiety and depression among Korean students increased. Compared to people who regularly practice sports, people with a predominantly sedentary lifestyle are more susceptible to stress [28].

This study aimed to assess the coping strategies with stress among medical students in Poland during the COVID-19 pandemic. We examined methods and styles of coping with stressful situations at the beginning of the COVID-19 pandemic, six months later and one year from its commencement using the same measures deployed in representative national surveys before the COVID-19 pandemic began. We also aimed to assess the factors associated with feelings of stress and failure during and at the beginning of the COVID-19 pandemic, such as alcohol intake and tobacco smoking. Therefore, the prevalence of alcohol consumption and smoking and their intensity at the beginning of the COVID-19 pandemic and after one year were examined. Additionally, the level of physical activity in the examined population, as one of the coping strategies with stress, was assessed.

## 2. Materials and Methods

### 2.1. Characteristics of the Studied Group at Three Analyzed Stages

The research was conducted in a group of students at the Wroclaw Medical University in the period from March 2020 to March 2021. These periods included the prevailing lockdown in Poland, where most of the time all social meeting points were fully or partially excluded from use. These periods did not differ in terms of the applied restrictions. We conducted a cross-sectional study. The study enrolled 2920 adults (930 subjects from urban areas up to 100,000 inhabitants, 1154 subjects from urban areas more than 100,000 inhabitants and 836 subjects from partially rural areas; place of residence of students was an incidental aspect of where they come from or currently live). Three stages were distinguished in the study population: Stage I (*n* = 1320)—analyzed at the beginning of the pandemic (March 2020); Stage II (*n* = 845)—analyzed after six months of the pandemic (October 2020); Stage III (*n* = 755)—analyzed after one year of the pandemic (March 2021). It was one group of students, who completed the survey three times at intervals (Stage I, II and III). The differences in numbers of students in each stage are due to some of the respondents not completing the questionnaire every time. 64.02% of the initial group of study participants volunteered for Stage II, and 57.20% of the initial group reported to Stage III. Additionally, student enrollment procedures did not differ depending on the time of the study. Characteristics of the research group are presented in Table 1.

### 2.2. Coping Orientation to Problems Experienced Questionnaire

The Mini Coping Orientation to Problems Experienced questionnaire (Mini-COPE) was used, adapted by Juczyński and Ogińska-Bulik [29,30]. The Polish version of the Mini-COPE inventory consists of 28 statements that make up 14 strategies for coping with stress. The tool is used to assess typical responses in situations of severe stress. The test indicates how often particular strategies are used when the subject is in a very difficult situation. The test is divided into four categories and the corresponding strategies (inventory scales): active coping (including active coping, planning, positive reappraisal), helplessness (including use of psychoactive substances, suppression of activities, self-blame), support (including seeking emotional support, seeking instrumental support), avoidance behaviors (including dealing with something else, denial, venting of emotions). Three strategies create independent factors (turning to religion, acceptance, sense of humor). The range of results for each statement is from 0 to 3 points. The numbers mean: 0 = “I hardly ever do this”, 1 = “I rarely do it”, 2 = “I do it often”, 3 = “I almost always do it”. The results were calculated by adding up the points for the answers to the two statements on the scale and then divided by two.

### 2.3. The Alcohol Use Disorders Identification Test 

 Alcohol use disorders were diagnosed with the WHO’s Alcohol Use Disorders Identification Test (AUDIT) [31]. This test is used as a screening tool to identify people whose alcohol consumption has become hazardous, harmful or addictive. The method is based on multi-country studies which show that certain symptoms (e.g., high alcohol consumption, alcohol-related violation of social norms, alcohol-related injuries) can be early warning signs of an alcohol problem and developing an addiction. As an attempt to identify symptoms that would be common to different countries and cultures, the test consists of three parts related to the amount and frequency of drinking (questions no. 1–3), to alcohol dependence (questions no. 4–6), and four questions on problems caused by alcohol (questions no. 7–10). The scoring based on these ten questions was as follows: 8–15 points indicate probable hazardous drinking, 16–19 points indicate harmful drinking, and 20 points or more probable alcohol dependence [31].

### 2.4. The Fagerström Test for Nicotine Dependence

The probability of whether a person is pharmacologically addicted to nicotine was assessed using a Fagerström’s questionnaire [32,33]. Pharmacological dependence implies not only a habit but also a biological need to get nicotine into the body. The Fagerström Nicotine Tolerance Test consists of six questions, each of which ranges from 0 to 1 or 0 to 3. Of all the questions, the most important one is “How soon after waking do you smoke your first cigarette?”. In order to assess the level of addiction to nicotine, all the questions are answered and then the values assigned to the answers are added up. Obtaining 0–4 points indicates no pharmacological addiction to nicotine or a very “low” level. Rather, smoking is a habit that a person does not want or cannot get rid of. Obtaining 5–8 points indicates the presence of features of nicotine addiction. It is difficult for such a person to do without a cigarette, especially in stressful situations or following an example (under pressure, at the instigation of the environment). Obtaining 9–11 points clearly indicates the presence of symptoms of pharmacological nicotine addiction, and some illnesses and discomforts that are undoubtedly related to smoking.

### 2.5. International Physical Activity Questionnaire

 In each of the study groups, analysis of physical activity was carried out using the methods of diagnostic survey with the use of the international physical activity questionnaire (IPAQ). The short form (IPAQ-S) was used to monitor health risks and to assess the level of physical activity. This version consists of seven questions concerning a period of one week, in which moderate and high-intensity efforts as well as walking and sitting are performed. The data obtained from the IPAQ-S questionnaire enabled us to classify the population into three groups: inactive (low level), moderately active (moderate level) and very active (high level) [34] or as metabolic equivalent of task (MET) value minutes a week. MET minutes represent the amount of energy expended carrying out physical activity; it is an equivalent of resting metabolism equal to energy expenditure corresponding to consumption of 3.5 mL of oxygen per kilogram of body weight per minute. There are three categories in which the MET minutes can be used (moderate activity, vigorous activity and walking). The sedentary time is shown in minutes. The questionnaire assessed the intensity of the physical activity in agreement with the following MET values: 3.3 MET—low intensity, 4.0 MET—moderate intensity, and 8.0 MET—high intensity [35].

### 2.6. Procedures

The participants of this study were students taking compulsory physical education classes once a week. Each examined stage received a link on the day of students’ classes (Stage I in March 2020, Stage II in October 2020, Stage III in March 2021) to complete anonymous questionnaires. For the filling of the questionnaire, each research participant had one didactic week (7 days). The respondents were asked to fill in questionnaires sent via the Internet using the Google documents tool. It is a platform for online file transfer and collaborative editing. The respondents received a link redirecting them to the online questionnaire placed in the Google documents tool. The files were questionnaires of the relevant tests that the students could complete over the Internet. The order of completing the tests was as follows: the Mini Coping Orientation to Problems Experienced questionnaire, Alcohol Use Disorders Identification Test, The Fagerström Test for Nicotine Dependence, International Physical Activity Questionnaire. The questionnaires were completed in Polish and the English version is available in Supplementary Materials (Questionnaire S1, S2, S3 and S4). The data concerning material status, place of residence, the field of study and anthropometric parameters: body weight, body height, as well as questions about feelings and behavior during the COVID-19 pandemic, were taken into account. Anthropometric data such as weight and height were self-reported by the respondents because it was not possible to collect this data in any other way due to the prevailing pandemic conditions. Participation in research was voluntary, anonymous, and had the approval of the Bioethics Committee at the Wroclaw Medical University (No. KB-251/2020). 

### 2.7. Statistical Analyses of Data

The analyses were carried out using the STATISTICA 13.3 (Statsoft Polska, Sp. z o.o.) package under Wroclaw Medical University’s license. The normality of the distribution of variables was checked using the Kołmogorov–Smirnov test, and the homogeneity of variance using Brown–Forsythe’s test because the size of each stage was different (Tables S1 and S2). To compare two independent variables with each other the chi-square test was used with Bonferroni correction. In order to test statistically significant differences between groups the non-parametric Skillings–Mack test with the post-hoc Dunn’s test (for variables not meeting the conditions of normal distribution) was used. Spearman’s rank order test was used for correlation analysis. Statistical significance was assumed for *p* < 0.05.

## 3. Results

### 3.1. The Stress-Coping Methods Used during the COVID-19 Pandemic

The analysis revealed statistically significant differences in stress-coping methods at the beginning of the pandemic (Stage I) and after six months of the COVID-19 pandemic (Stage II). After six months of the pandemic, it was shown that, compared to the stage at its beginning, there was a decrease in the frequency of matching the following methods: positive reframing, acceptance, sense of humor and self-distraction (Table 2). However, the use of stress-coping methods, such as active stress-coping, planning, seeking emotional and instrumental support, denial, venting of emotions, use of psychoactive substances, suppression of activities and self-blame were more often used after six months of pandemic compared to the stage at its beginning (Table 2). Similar results were shown when the stage after one year of the pandemic of COVID-19 was compared to the stage from its beginning (Table 2). The changes in the frequency of matching the stress-coping methods between the stages after six months and after one year of the pandemic were not statistically significant (Table S3 in Supplementary Materials).

The correlations between stress-coping methods and age in each of the examined stages of the pandemic were shown (Table S4 in Supplementary Materials).

### 3.2. Alcohol Use Disorders Identification Test during the Pandemic

The analysis of the results for the Alcohol Use Disorders Identification Test (AUDIT) questionnaire showed that 845 individuals included in Stage I were qualified as alcohol users. For Stages II and III, the number of alcohol users was 705 and 684, respectively. 

It was shown that after six months of the pandemic the distribution of alcohol users was significantly changed (Table 3). It was shown that during Stage II, there was a decreased number of individuals characterized by a very low risk of addiction to alcohol (AUDIT score <8 points). However, the percentage of subjects with a probability of hazardous and harmful drinking and of alcohol dependence significantly increased. Similar changes were observed during Stage III in comparison to Stage I (Table 3). A difference in the level of alcohol dependence between Stages II and III was also observed (Table S3 in Supplementary Materials). A smaller number of individuals with a low risk of alcohol dependence in Stage III compared to Stage II was shown (*p* = 0.0011). However, in the group of alcohol users with a probability of hazardous dependence (AUDIT score 8–15 points), there was an increase in the frequency of alcohol use after one year of COVID-19 pandemic (Stage III) compared to alcohol drinking after six months of the pandemic (Stage II). A similar change in alcohol drinking was noted in the group of alcohol abusers with likely alcohol dependence (AUDIT score > 20 points). Interestingly, in the group of alcohol abusers displaying harmful drinking (AUDIT score 16–19 points), no difference was shown in the frequency of drinking after six months of the pandemic (Stage II) compared to after one year of the pandemic (Stage III) (Table S5 in Supplementary Materials).

### 3.3. The Intensity of Smoking during the COVID-19 Pandemic

The analysis of the results for Fagerström’s nicotine addiction questionnaire showed that 82 individuals included in Group I were qualified as smokers. For Stages II and III, the numbers of smokers were 71 and 98, respectively.

During Stage I (analyzed at the beginning of the pandemic, treated as a point of reference), no smokers with the features and clear symptoms of nicotine addiction were found among students (5–8 points and 9–11 points in Fagerström’s questionnaire, respectively) (Table 4). After six months of the pandemic (Stage II), changes in the intensity of smoking were not shown but after one year of the pandemic (Stage III), it was observed that the percentage of smokers without pharmacological or biological addiction to nicotine (0–4 points in Fagerström’s questionnaire) was lower compared to the reference group (Stage I). Additionally, it was found that the percent of smokers with features of nicotine addiction (5–8 points in Fagerström’s questionnaire) after one year of pandemic (Stage III) was significantly higher compared to the results observed before the pandemic (Stage I) (Table 4).

There was also a difference in the intensity of smoking after six months of the pandemic (Stage II) and after one year of the pandemic (Stage III). After one year of the pandemic, the percentage of smokers without pharmacological nicotine addiction (0–4 points in Fagerström’s questionnaire) was found to be higher compared to the results obtained six months earlier. It was not shown that the percentage of smokers with the symptoms of nicotine addiction after one year of pandemic changed compared to the percentage of smokers after six months of pandemic (smokers with 5–8 and 9–11 points in Fagerström’s questionnaire, respectively) (Table S6 in Supplementary Materials).

### 3.4. Physical Activity during the COVID-19 Pandemic

The analysis of the results of IPAQ showed that the physical activity of subjects was gradually decreasing during the COVID-19 pandemic (Table 5). Significant differences between the number of people engaging in low-, medium- and high-intensity exercise during Stage II (after six months of the pandemic) and Stage III (after one year of the pandemic) in comparison to the beginning of the COVID-19 pandemic (Table 6) were noted. No changes in the intensity of physical activity were observed between Stage II and III in the group of people engaging in low-, medium- and high-intensity exercise (Tables S7 and S8 in Supplementary Materials).

## 4. Discussion

### 4.1. Coping with Stress during the Pandemic

One of the aims of this study was to investigate the changes in the ways in which medical students dealt with stress over the course of the COVID-19 pandemic. The stressor related with this situation that affects us is a completely new phenomenon, so there is a need to diagnose the level of stress during a pandemic and to introduce measures to reduce its effects for the health of individuals. It was noted that six months after the onset of the pandemic, compared to the beginning stage, a decrease in the frequency of positive reappraisal, acceptance, sense of humor and dealing with something else were observed. These results correspond with the results of other studies that have focused on determining the impact of a pandemic on mood changes and emotions [36,37,38,39,40,41]. This is due to the fact that, in its first meaning, stress as a stimulus refers to a situation inducing tension and strong emotions in an individual to encourage activities aimed at dealing with stress. For example, nursing students face multiple new and difficult situations, which can be described as stressful events, in the clinical process of improving their professional skills acquired during programme-related courses [42,43]. The intensity of the stress experienced in the clinical environment is primarily related to the care of the patient, accompanying another person in the face of death, performing nursing procedures and the burden of negative interactions with the staff and lecturers [44,45]. Spanish studies have confirmed that clinical stressors are perceived more intensively compared to academic and external stressors [46,47]. The stress experienced and remedial activities undertaken by students modify their quality of life and health. The high levels of stress can lead to serious health issues [48].

Strategies of coping with stress, such as active coping, planning, seeking emotional and instrumental support, denial, venting of emotions, use of psychoactive substances, suppression of activities, self-blame, were used more often after six months of pandemic compared to its initial stage. Similar results were obtained when the stage after one year of the COVID-19 pandemic and the one before its onset were studied. This is not particularly surprising because the American Psychiatric Association (APA) estimates that the negative impact of the situation caused by SARS-CoV-2 on the human psyche will be observed in nearly 50% of the population [49]. The symptoms may appear even several months after lifting the most severe restrictions [50]. Chodkiewicz et al. [37] indicated that the COVID-19 pandemic has serious consequences for mental health and coping with stress. The respondents with significantly reduced mental well-being use maladaptive coping strategies, such as denial of problems, emotional discharge, taking in psychopharmaceuticals, doing nothing and self-blaming. Similar results were obtained in physiotherapy students, where the main strategies for coping with stress were positive re-evaluation, discharging, self-blaming and taking psychopharmaceuticals [51]. Another report also indicated that younger adults (like students) were under greater stress during the pandemic. A study conducted on Polish students from various universities showed that students most often chose such strategies of coping with stress as acceptance, planning and seeking emotional support. The results also showed that the youngest students coped with stress the worst [52]. These studies show how difficult it was for students to adapt to the new situation caused by the pandemic. For this particular group, not only the social life, but also the functioning of the university changed, which resulted in the necessity to adapt to a completely new mode of education.

### 4.2. Alcohol Consumption and Cigarette Smoking during the Epidemic

One of the ways to deal with stress and emotions is through the abuse of any type of stimulant, including alcohol. A reflection of this statement can be found in the results of this study. It has been shown that after six months of the pandemic a reduction in the number of persons with a very low risk of alcohol dependence was observed. On the other hand, the percentage of people with probable risky and harmful drinking and probable alcohol dependence increased significantly. Similar changes were observed after one year of the pandemic as compared to the beginning of the pandemic. In the group of people abusing alcohol who display harmful drinking behavior, there was no difference in the frequency of drinking after six months of the pandemic compared to that after a year of the pandemic. Similar results have been observed in studies conducted in many countries around the world over a similar period [37,48,53,54,55]. The drinking of alcohol can be one of the ways of coping with stress, regulating tension and also avoiding confrontation with problems [55]. As evidenced in mammalians, long isolation results in increased stress levels, while alcohol is one of the psychoactive substances that can be used to reduce stress [56]. It should also be noted that in our study we assessed alcohol consumption in a specific social group, namely medical students. This group is extremely vulnerable to stress, and alcohol is a fairly cheap and simple way to deal with emotions. Alcohol consumption during the epidemic in different social groups, including students, is the subject of many scientific reports [55]. As it turns out, similar results were obtained by other authors who also analyzed medical students [55,57,58]. 

From the analysis of the available literature, it can be concluded that during the COVID pandemic, the manner of alcohol consumption and amount of alcohol consumed changed. An interesting observation may be that there are different scenarios of alcohol consumption during the pandemic, as evaluated by Rehm et al. in their study [59]. They found two scenarios of alcohol consumption behavior during the COVID-19 pandemic: the first scenario predicts an increase, while the second one predicts a decrease in alcohol consumption [60]. This fact can be confirmed by the results of our own research, where we observed a higher percentage of people drinking alcohol and also those who display risky or harmful drinking behavior and indicative of addiction. It is consistent with the result showed by Koopmann et al. [61] and Grossman et al. [62]. Fear and anxiety related to the duration of the epidemic may be factors that cause the increase of alcohol consumption [19,61]. Reasons for increased drinking also included increased alcohol availability and boredom as reported by Grossman et al. [62]. Increased consumption may be related to the psychological effect of the coronavirus pandemic and social constraints [60]. 

 Smoking is, next to alcohol, the most common stimulant. As it turned out, some people in response to stress may also smoke cigarettes more often. Our study found that the greatest increase in smokers occurred after one year of the pandemic, when the percentage of smokers doubled compared to the pre-pandemic period, what may be a result of stress symptoms increasing as a consequence of long-term social isolation resulting from the ongoing pandemic. The COVID-19 pandemic can be considered to be a stressful event, and it can be expected that individuals under nationwide lockdown are facing, at least temporarily, higher stress levels due to the need to adapt to the prolonged stay at home as reported earlier [56]. This would be in line with the results of other studies where, over the course of the pandemic, an increase in the number of people who smoked cigarettes was observed. Carreras et al. [63] and Koopmann et al. [61] reported that increased cigarette consumption can be related to increased mental distress. In the study by Carreras et al. [63], lockdown caused an increase in cigarette consumption by 9.1%, and was most commonly associated with deterioration in quality of life, decreased sleep, increased anxiety and depressive symptoms. In turn, in the study by Koopmann et al. [61], almost half of the participants (45.8%) increased the amount of smoking during the pandemic. In addition, the same study also saw an increase in alcohol consumption during lockdown (35.5% of participants reported an increase in drinking). This may indicate that the coronavirus pandemic in general, through stress, contributed to an increase in the frequency of use of stimulants. 

### 4.3. Physical Activity during the Pandemic

The role of physical activity for health is indisputable. There are a number of studies confirming that the level of physical activity has an impact on mood and well-being [39,40,41,64]. Changes in physical activity may also be related to the current epidemiological situation, not only due to the lack of access to certain activities, but also due to increased stress. Our study showed that the physical activity of the subjects gradually decreased during the COVID-19 pandemic. There were significant differences between the number of people performing low, medium-, and high-intensity exercise after six months and after one year of the pandemic compared with the beginning of the pandemic. There was no statistically significant change in physical activity intensity between Stages II and III of the pandemic in the low-, medium-, and high-intensity exercise groups. This may result from the restrictions and social moods prevailing at that time. With the implementation of the restrictions on social isolation in March 2020, customary places of physical activity such as gyms, sports centres, swimming pools and outdoor recreational facilities were no longer available. Some individuals may have sufficient self-regulation of physical activity to pursue alternative activities (e.g., online fitness classes or other home-based physical activities), while others have reduced their physical activity as a result of the pandemic due to the lack of social support or fear of contracting the virus in an outdoor environment. On the other hand, it should be noted that people who are forced to work from home or do remote activities could spend less time commuting to work or university and could take the opportunity to develop new habits of physical activity. Similar results were observed in Srivastav’s study conducted during the lockdown in March and April 2020 on physiotherapy students in India, where they found a 48% reduction in activity level and 49% reduction in energy expenditure compared to before the outbreak [65]. Other studies conducted on college students during the pandemic also found reduced levels of physical activity. Changes in the number of minutes spent walking and sitting were also observed [66].

Moreover, the lowest amount of time spent walking in the study group was recorded after six months of the pandemic. The amount of time spent sitting during the day varied. The students spent the lowest amount of time sitting in October 2020, which was six months after the outbreak. This may have been related to the start of the academic year and the implementation of classes in a hybrid form (via Internet and stationary form) and not just remotely as previously. Similar changes were observed by other researchers where they also assessed the levels of walking and staying seated using the IPAQ questionnaire [22,67]. The limitation and reduction of physical activity level during the lockdown and ongoing pandemic is undeniable as confirmed by numerous reports. However, it should be noted that there are reports that do not confirm this finding [68,69,70].

 In fact, during lockdown, people modified their lifestyles, with an increase in sedentary time as people spent more time at home, and a decrease in physical activity [71]. In contrast to health-promoting behaviors such as physical activity, some people may cope with social isolation and pandemic-related psychological stress by engaging in or intensifying adverse health behaviors such as smoking or alcohol drinking [58].

## 5. Limitations of the Study

Presented studies have shown changes in the key outcomes to be COVID-19-related. However, our results were only of a cross-sectional and informative character. The study group consisted of a narrow group of Polish society (students of medicine). Moreover, the individuals selected to the study group had no chronic metabolic diseases or psychological disease. On the other hand, the studies conducted on this group are difficult to compare with other results because of poor information in literature about similar studies focused on young people. Because of the above, the effect of COVID-19 on the studied behavioral factors cannot be extrapolated to the adult human population. Therefore, it seems reasonable to continue the research on an enlarged group by other social subpopulation, in order to expand the results obtained in this study. 

## 6. Conclusions

The COVID-19 pandemic has exacerbated stress and fear among medical students, and long-term isolation resulted in worse coping with stress. This, in turn, undoubtedly influenced the increase in the frequency of unhealthy behaviors such as alcohol consumption and smoking. Additionally, the transfer of most spheres of life to the Internet, including learning and spending free time, contributed to the reduction of physical activity in favor of a sedentary lifestyle.

## Figures and Tables

**Table 1:** Characteristic of the study group.

Stage Parameter	Stage I	Stage II	Stage III
Sex Women/Men %women/%men	1007/313 76.29%/23.71%	632/213 74.79 %/25.21%	599/156 79.34%/20.66%
Age [years] X ± SD	20.97 ± 2.15	20.13 ± 1.55	20.45 ± 1.87
BMI [kg/m^2^]	21.95 ± 4.76	21.43 ± 2.92	21.89 ± 3.34

Legend: X—average; SD—standard deviation.

**Table 2:** Association between coping with stress and progression of COVID-19 pandemic.

Stage Strategy	Stage I *		Stage II				Stage III		
	Median	X ± SD	Median	X ± SD	*p* ^1^	*p* ^2^	Median	X ± SD	*p* ^2^
Active stress-coping methods	2.00	1.75 ± 0.73	2.00	2.15 ± 0.76	**<0.0001**	**<0.0001**	2.00	2.08 ± 0.81	**<0.0001**
Planning	2.00	1.82 ± 0.73	2.00	2.04 ± 0.80	**<0.0001**	**<0.0001**	2.00	2.01 ± 0.83	**<0.0001**
Positive re-evaluation	2.00	1.61 ± 0.78	1.50	1.43 ± 0.86	**0.0000**	**<0.0001**	1.50	1.47 ± 0.91	**0.0010**
Acceptance	2.00	2.01 ± 0.68	2.00	1.72 ± 0.80	**<0.0001**	**<0.0001**	2.00	1.67 ± 0.82	**<0.0001**
Sense of humor	1.00	1.02 ± 0.67	1.00	0.95 ± 0.71	**0.0030**	**0.0020**	1.00	0.92 ± 0.73	**0.0000**
Turning to religion	0.50	0.86 ± 0.96	0.50	0.92 ± 1.00	0.1720	-	0.50	0.95 ± 1.02	-
Seeking emotional support	2.00	1.82 ± 0.82	2.00	1.99 ± 0.90	**<0.0001**	**<0.0001**	2.00	1.93 ± 0.93	**<0.0001**
Seeking instrumental support	2.00	1.63 ± 0.82	2.00	1.88 ± 0.93	**<0.0001**	**<0.0001**	2.00	1.87 ± 0.90	**<0.0001**
Dealing with other things	2.00	1.71 ± 0.71	1.50	1.56 ± 0.76	**<0.0001**	**<0.0001**	1.50	1.55 ± 0.78	**<0.0001**
Denial	2.00	1.75 ± 0.73	2.00	2.15 ± 0.76	**0.0370**	**<0.0001**	2.00	2.08 ± 0.81	**<0.0001**
Giving vent to one’s feelings	2.00	1.82 ± 0.73	2.00	2.04 ± 0.80	0.0800	-	2.00	2.01 ± 0.83	-
Taking in psychopharmaceuticals	2.00	1.61 ± 0.78	1.50	1.43 ± 0.86	**0.0140**	**0.0010**	1.50	1.47 ± 0.91	**<0.0001**
Doing nothing	2.00	2.01 ± 0.68	2.00	1.72 ± 0.80	0.2490	-	2.00	1.67 ± 0.82	-
Self-blaming	1.00	1.02 ± 0.67	1.00	0.95 ± 0.71	**<0.0001**	**<0.0001**	1.00	0.92 ± 0.73	**<0.0001**

Legend: * Stage I—treated as a point of reference for further analysis, Stage I was compared with Stage II and Stage III; X—average; SD—standard deviation; *p*—significance level; ^1^—values obtained in the Skillings–Mack test; ^2^—values obtained in the post-hoc Dunn’s test. Data were obtained with the Skillings–Mack test and the Dunn’s test. *p*-values marked with bold indicate statistically significant differences between the groups.

**Table 3:** Association between alcohol drinking pattern and progression of COVID-19 pandemic.

Stage Number of Points	Stage I *		Stage II							Stage III						
	N	%	N	%	χ2	OR	95% CI	*p*	Bonferroni Correction	N	%	χ2	OR	95% CI	*p*	Bonferroni Correction
<8 points	772	91.36%	554	78.58%	66.14	2.43	1.96–3.03	**<0.0001**	0.0042	470	68.71%	110.88	0.25	0.19–0.32	**<0.0001**	0.0042
8–15 points	66	7.81%	133	18.87%	131.00	6.84	4.82–9.70	**<0.0001**	0.0042	189	27.63%	296.13	0.05	0.04–0.08	**<0.0001**	0.0042
16–19 points	4	0.47%	8	1.13%	10.26	6.79	2.01–22.87	**0.0014**	0.0042	11	1.61%	37.88	0.05	0.02–0.18	**<0.0001**	0.0042
>20 points	3	0.36%	10	1.42%	17.67	11.31	3.07–41.66	**<0.0001**	0.0042	14	2.05%	56.56	0.03	0.01–0.11	**<0.0001**	0.0042

Legend: * Stage I—treated as a point of reference for further analysis, Stage I was compared with Stage II and Stage III; χ2—“χ2 score” in chi square test; OR—odds ratio; 95% CI—confidence interval; *p*—significance level. Data were obtained with the chi square test. *p*-values marked with bold indicate statistically significant differences between the groups.

**Table 4:** Association between smoking pattern and progression of COVID-19 pandemic.

Stage Number of Points	Stage I *		Stage II							Stage III						
	N	%	N	%	χ2	OR	95% CI	*p*	Bonferroni Correction	N	%	χ2	OR	95% CI	*p*	Bonferroni Correction
Non-smoker	1238	93.79%	774	92.69%	1.60	1.22	0.90–1.67	0.2066	0.0042	657	87.02%	16.75	1.86	1.38–2.50	**<0.0000**	0.0042
0–4 points	82	6.21%	54	6.47%	0.45	0.89	0.62–1.26	0.5024	0.0042	93	12.32%	28.98	2.32	1.69–3.16	**<0.0001**	0.0042
5–8 points	0	0%	6	0.72%	7.71	–	–	0.0055	0.0042	4	0.53%	5.38	–	–	0.0204	0.0042
9–11 points	0	0%	1	0.12%	0.07	–	–	0.7854	0.0042	1	0.13%	0.13	–	–	0.7161	0.0042

Legend: * Stage I—treated as a point of reference for further analysis, Stage I was compared with Stage II and Stage III; χ2—“χ2 score” in chi square test; OR—odds ratio; 95% CI—confidence interval; *p*—significance level. Data were obtained with the chi square test. *p*-values marked with bold indicate statistically significant differences between the groups.

**Table 5:** Association between physical activity and progression of COVID-19 pandemic.

Stage The Intensityof Effort	Stage I *		Stage II							Stage III						
	N	%	N	%	χ2	OR	95% CI	*p*	Bonferroni Correction	N	%	χ2	OR	95% CI	*p*	Bonferroni Correction
Low	786	59.59	631	74.68	54.56	0.49	0.40–0.59	**<0.0001**	0.0056	580	76.82	63.73	0.44	0.36–0.54	**<0.0001**	0.0056
Medium	305	23.12	108	12.78	36.04	0.49	0.38–0.62	**<0.0001**	0.0056	90	11.92	44.65	0.42	0.32–0.54	**<0.0001**	0.0056
High	228	17.29	106	12.54	8.83	0.69	0.54–0.88	**0.0030**	0.0056	85	11.26	10.60	0.65	0.50–0.84	**0.0011**	0.0056

Legend: * Stage I—treated as a point of reference for further analysis, Stage I was compared with Stage II and Stage III; χ2—“χ2 score” in chi square test; OR—odds ratio; 95% CI—confidence interval; *p*—significance level. Data were obtained with the chi square test. *p*-values marked with bold indicate statistically significant differences between the groups.

**Table 6:** Association between physical activity (broken down by exercise intensity (Low, Medium, High) and type of physical activity) and progression of COVID-19 pandemic.

				Part A				
Type of Physical Activity		Vigorous Physical Activity		Moderate Physical Activity		Walking	Sedentary Time	
Together		Q = 114.77, ***p* < 0.0001**		Q = 44.19, ***p* < 0.0001**		Q = 28.49, ***p* < 0.0001**	Q = 61.01, ***p* < 0.0001**	
Low		Q = 7.17, ***p* = 0.028**		Q = 4.52, *p* = 0.104		Q = 30.91, ***p* < 0.0001**	Q = 39.38, ***p* < 0.0001**	
Medium		Q = 2.04, *p* = 0.360		Q = 7.05, ***p* = 0.030**		Q = 17.70, ***p* = 0.000**	Q = 17.66, ***p* = 0.000**	
High		Q = 0.77, *p* = 0.681		Q = 0.22, *p* = 0.896		Q = 5.47, *p* = 0.065	Q = 11.91, ***p* = 0.003**	
**“Low”, “Medium” and “High” together**								
**Vigorous Physical Activity** **[MET]—Median**			**Moderate Physical Activity** **[MET]—Median**		**Walking** **[MET]—Median**			**Sedentary Time [MET]—Median**
Stage I—480, Stage II—320 ***p* = 0.001**		Stage I—360, Stage II—240 ***p* = 0.001**			Stage I—462, Stage II—693 ***p* < 0.0001**		Stage I—360, Stage II—300 ***p* < 0.0001**	
**Low**		***p***	**Medium**		***p***	**High**		***p***
**Vigorous physical activity [MET]—Median**			**Vigorous physical activity [MET]—Median**			**Vigorous physical activity [MET]—Median**		
**Stage I**	**Stage II**		**Stage I**	**Stage II**		**Stage I**	**Stage II**	
450	420	0.375	720	960	-	2160	2160	-
**Low**		***p***	**Medium**		***p***	**High**		***p***
**Moderate physical activity [MET]—Median**			**Moderate physical activity [MET]—Median**			**Moderate physical activity [MET]—Median**		
**Stage I**	**Stage II**		**Stage I**	**Stage II**		**Stage I**	**Stage II**	
160	160	-	490	280	**0.004**	480	480	-
**Low**		***p***	**Medium**		***p***	**High**		***p***
**Walking [MET]—Median**			**Walking [MET]—Median**			**Walking [MET]—Median**		
**Stage I**	**Stage II**		**Stage I**	**Stage II**		**Stage I**	**Stage II**	
396	676.5	**<0.0001**	462	693	**0.012**	693	924	-
**Low**		***p***	**Medium**		***p***	**High**		***p***
**Sedentary time [min]—Median**			**Sedentary time [min]—Median**			**Sedentary time [min]—Median**		
**Stage I**	**Stage II**		**Stage I**	**Stage II**		**Stage I**	**Stage II**	
360	300	**<0.0001**	360	240	**<0.0001**	360	300	**0.002**
**Part C**								
**“Low”, “Medium” and “High” together**								
**Vigorous Physical Activity** **[MET]—Median**			**Moderate Physical Activity** **[MET]—Median**		**Walking** **[MET]—Median**			**Sedentary Time [MET]—Median**
Stage I—480, Stage III—160 ***p* < 0.0001**		Stage I—360, Stage III—160 ***p* < 0.0001**			Stage I—462, Stage III—792 ***p* < 0.0001**		Stage I—360, Stage III—240 ***p* = 0.000**	
**Low**		***p***	**Medium**		***p***	**High**		***p***
**Vigorous physical activity [MET]—Median**			**Vigorous physical activity [MET]—Median**			**Vigorous physical activity [MET]—Median**		
**Stage I**	**Stage III**		**Stage I**	**Stage III**		**Stage I**	**Stage III**	
450	480	0.518	720	720	-	2160	1920	-
**Low**		***p***	**Medium**		***p***	**High**		***p***
**Moderate physical activity [MET]—Median**			**Moderate physical activity [MET]—Median**			**Moderate physical activity** **[MET]—Median**		
**Stage I**	**Stage III**		**Stage I**	**Stage III**		**Stage I**	**Stage III**	
160	90	-	490	240	**0.001**	480	680	-
**Low**		***p***	**Medium**		***p***	**High**		***p***
**Walking [MET]—Median**			**Walking [MET]—Median**			**Walking [MET]—Median**		
**Stage I**	**Stage III**		**Stage I**	**Stage III**		**Stage I**	**Stage III**	
396	693	**<0.0001**	462	792	**<0.0001**	693	1386	-
**Low**		***p***	**Medium**		***p***	**High**		***p***
**Sedentary time [min]—Median**			**Sedentary time [min]—Median**			**Sedentary time [min]—Median**		
**Stage I**	**Stage III**		**Stage I**	**Stage III**		**Stage I**	**Stage III**	
360	240	**0.001**	360	300	**0.239**	360	300	**0.240**

Legend: *p*—significance level. In part A data were obtained with the Skillings–Mack test. In parts B and C data were obtained with the post-hoc Dunn’s test. *p*-values marked with bold indicate statistically significant differences between the groups.

## Data Availability

The data are not publicly available to protect participants’ privacy.

## Supplementary Materials

The following are available online at https://www.mdpi.com/article/10.3390/ijerph19010302/s1, Questionnaire S1. Coping Orientation to Problems Experienced questionnaire (Mini-COPE), Questionnaire S2. The Alcohol Use Disorders Identification Test (AUDIT), Questionnaire S3. The Fagerström Test for Nicotine Dependence, Questionnaire S4. International Physical Activity Questionnaire—Short Form (IPAQ-S), Table S1. The results of Kołmogorow–Smirnow’s test and Brown-Forsythe’s test for strategies of coping with stress, Table S2. The results of Kołmogorow–Smirnow’s test and Brown–Forsythe’s test for types of physical activity, Table S3: Association between coping with stress and progression of COVID-19 pandemic, Table S4: The correlations between stress-coping methods with age in each of the examined stages of COVID-19 pandemic, Table S5: Association between alcohol drinking pattern and progression of COVID-19 pandemic, Table S6: Association between smoking pattern and progression of COVID-19 pandemic, Table S7: The physical activity after six months and after one year of COVID-19 pandemic, Table S8: The physical activity after six months and after one year of COVID-19 pandemic.
