# Supplementary material for: The Changes in Stress Coping, Alcohol Use, Cigarette Smoking and Physical Activity during COVID-19 Related Lockdown in Medical Students in Poland
Source: Int J Environ Res Public Health. 2021 Dec 28;19(1):302. doi: 10.3390/ijerph19010302 (PMC8744869; doi:10.3390/ijerph19010302)
Supplement: Supplementary file 1 [file ijerph-19-00302-s001.zip › ijerph-1497003-supplementary.pdf]

Supplementary Materials

Questionnaire S1. Coping Orientation to Problems Experienced questionnaire (Mini-COPE).

Charles Carver

Mini-COPE

Adaptation: Zygfryd Juczyński, Nina Ogińska-Bulik

.... Age: .... Sex: M/K Date: ....

People react differently when they encounter difficult or stressful events in their lives. You can deal with stress advise in a variety of ways. The questionnaire is designed to establish how you usually behave when you experience these events. It is understandable that different events trigger different reactions, but what do you usually do when you are experiencing a lot unpleasant event? In each line, mark one answer that suits you best, circling it around the appropriate digit (0-1-2-3). Do not omit any statement.

The individual numbers mean: 0 = "I hardly ever do this", 1 = "I rarely do this", 2 = "I do this often", 3 = "I almost always do this".

When I am in a very difficult situation, it is usually:

1. I do work or other activities so as not to think about it. 0 1 2 3
2. My efforts are focused on doing something about the situation. 0 1 2 3
3. I say to myself "that's not true". 0 1 2 3
4. I drink alcohol or take other measures to make me feel better. 0 1 2 3
5. I get emotional support from others. 0 1 2 3
6. I give up trying to achieve the goal. 0 1 2 3
7. I am taking action to improve the situation. 0 1 2 3
8. I don't want to believe this really happened. 0 1 2 3
9. I'm talking about things that help me get away from unpleasant feelings. 0 1 2 3
10. I am looking for advice and help from others on what to do. 0 1 2 3
11. I drink alcohol or take other measures to help me get through this. 0 1 2 3
12. I am trying to see it in a different, more positive light. 0 1 2 3
13. I criticize myself. 0 1 2 3
14. I am trying to develop a strategy or plan defining what to do. 0 1 2 3
15. I receive encouragement and understanding from others. 0 1 2 3

When I am in a very difficult situation, it is usually:

16. I give up on dealing with it. 0 1 2 3
17. I am looking for good points in what happened. 0 1 2 3
18. I'm just kidding about this. 0 1 2 3
19. I do something to think less about it, e.g. go to the cinema, watch TV, read, I am daydreaming, sleeping or shopping. 0 1 2 3
20. I accept that this has already happened. 0 1 2 3
21. I reveal my negative emotions. 0 1 2 3
22. I am trying to find solace in religion or in my faith. 0 1 2 3
23. I get help or advice from other people. 0 1 2 3
24. I'm learning to live with it. 0 1 2 3
25. I am seriously wondering what steps should be taken. 0 1 2 3
26. I blame myself for what happened. 0 1 2 3
27. I pray or meditate. 0 1 2 3
28. I treat this situation as playing. 0 1 2 3

Questionnaire S2. The Alcohol Use Disorders Identification Test (AUDIT).

AUDIT questionnaire

Please circle the answer that is correct for you

1. How often do you have a drink containing alcohol?

· Never
· Monthly or less
· 2-4 times a month
· 2-3 times a week
· 4 or more times a week

2. How many standard drinks containing alcohol do you have on a typical day when drinking?

· 1 or 2
· 3 or 4
· 5 or 6
· 7 to 9
· 10 or more

3. How often do you have six or more drinks on one occasion?

· Never
· Less than monthly
· Monthly
· Weekly
· Daily or almost daily

4. During the past year, how often have you found that you were not able to stop drinking once you had started?

· Never
· Less than monthly
· Monthly
· Weekly
· Daily or almost daily

5. During the past year, how often have you failed to do what was normally expected of you because of drinking?

· Never
· Less than monthly
· Monthly
· Weekly
· Daily or almost daily

6. During the past year, how often have you needed a drink in the morning to get yourself going after a heavy drinking session?

· Never
· Less than monthly
· Monthly
· Weekly
· Daily or almost daily

7. During the past year, how often have you had a feeling of guilt or remorse after drinking?

· Never
· Less than monthly
· Monthly
· Weekly
· Daily or almost daily

8. During the past year, have you been unable to remember what happened the night before because you had been drinking?

· Never
· Less than monthly
· Monthly
· Weekly
· Daily or almost daily

9. Have you or someone else been injured as a result of your drinking?

· No
· Yes, but not in the past year
· Yes, during the past year

10. Has a relative or friend, doctor or other health worker been concerned about your drinking or suggested you cut down?

· No
· Yes, but not in the past year
· Yes, during the past year

Scoring the AUDIT

Scores for each question range from 0 to 4, with the first response for each question (eg never) scoring 0, the second (eg less than monthly) scoring 1, the third (eg monthly) scoring 2, the fourth (eg weekly) scoring 3, and the last response (eg. daily or almost daily) scoring 4. For questions 9 and 10, which only have three responses, the scoring is 0, 2 and 4 (from left to right).

A score of 8 or more is associated with harmful or hazardous drinking, a score of 13 or more in women, and 15 or more in men, is likely to indicate alcohol dependence.

Saunders JB, Aasland OG, Babor TF et al. Development of the alcohol use disorders identification test (AUDIT): WHO collaborative project on early detection of persons with harmful alcohol consumption — II. Addiction 1993, 88: 791–803.

Questionnaire S3. The Fagerström Test for Nicotine Dependence.

Segment: _ _

Visit Number: _ _

Date of Assessment: (mm/dd/yyyy) _ _/_ _/_ _ _ _

Do you currently smoke cigarettes?

☐ No ☐ Yes

If "yes," read each question below. For each question, enter the answer choice which bestdescribes your response.

1. **How soon after you wake up do you smoke your first cigarette?**

   ☐ Within 5 minutes ☐ 31 to 60 minutes

   ☐ 6 to 30 minutes ☐ After 60 minutes

2. **Do you find it difficult to refrain from smoking in places where it is forbidden** (e.g., in church, at the library, in the cinema)**?**

   ☐ No ☐ Yes

3. **Which cigarette would you hate most to give up?**

   ☐ The first one in the morning ☐ Any other

4. **How many cigarettes per day do you smoke?**

   ☐ 10 or less ☐ 21 to 30

   ☐ 11 to 20 ☐ 31 or more

5. **Do you smoke more frequently during the first hours after waking than during the rest of the day?**

   ☐ No ☐ Yes

6. **Do you smoke when you are so ill that you are in bed most of the day?**

   ☐ No ☐ Yes

Comments:

Heatherton TF, Kozlowski LT Frecker RC (1991). The Fagerström Test for Nicotine Dependence: A revision of the Fagerström Tolerance Questionnaire. British Journal of Addiction 86:1119-27.

Instructions

Clinic personnel will follow standard scoring to calculate score based on responses.

Your score was: (your level of dependence on nicotine is): _ _

Heatherton TF, Kozlowski LT Frecker RC (1991). The Fagerström Test for Nicotine Dependence: A revision of the Fagerström Tolerance Questionnaire. British Journal of Addiction 86:1119-27.

Questionnaire S4. International Physical Activity Questionnaire – Short Form (IPAQ-S).

Evaluation Measures

YOUTHREX Research & Evaluation eXchange

International Physical Activity Questionnaire - Short Form

OVERVIEW

- This measure assesses the types of intensity of physical activity and sitting time that people do as part of their daily lives are considered to estimate total physical activity in MET-min/week and time spent sitting.

SUBSCALES

- None
- Sample items from the scale:
  - » During the last 7 days, on how many days did you do vigorous physical activities like heavy lifting, digging, aerobics, or fast bicycling?

STEPPING UP THEME(S) & OUTCOME(S)

- Health & Wellness
  - » Youth are physically healthy

TARGET POPULATION

- Youth 15 years of age and older

LENGTH & HOW IT IS MEASURED

- 7 items
- Open-ended questions surrounding individuals' last 7-day recall of physical activity
- Self-report, paper-pencil version or orally
- Available in: English and many other languages

DEVELOPER

- International Physical Activity Questionnaire, 1998

GOOD TO KNOW

- Used by the Ontario Trillium Foundation
- Click here for Guidelines for Data Processing and Analysis of the International Physical Activity Questionnaire (IPAQ) - Short Form

PSYCHOMETRICS

- Reliability

  Test-rest reliability indicated good stability

  High reliability (α <.80)

- Validity

  Predictive validity

  Concurrent validity

  Convergent validity

  Criterion validity

  Discriminant validity

LEARN MORE

- International Physical Activity Questionnaire. (2016).Home. Retrieved from https://sites.google.com/site/ theipaq/

- C.L. Craig, A. Marshall, M. Sjostrom, A. Bauman, M. Booth, B. Ainsworth, *et al*. International PhysicalActivity Questionnaire: 12-country reliability and validity. Med Sci Sports Exerc, 35 (2003), pp. 1381-1395

- Lee, P.H., Macfarlane, D.J., Lam, T.H., Stewart, S.M.(2011). *Validity of the international physical activity questionnaire short form (IPAQ-SF): A systematic review*. International Journal of Behavioral Nutrition and Physical Activity. 8:115.

- van Poppel MNM, Chinapaw MJM, Mokkink LB, van Mechelen W, Terwee CB: Physical activity questionnaires for adults: A systematic review of measurement properties. Sports Medicine. 2010, 40: 565-600. 10.2165/11531930-000000000- 00000.

(August 2002)

SHORT LAST 7 DAYS SELF-ADMINISTERED FORMAT

FOR USE WITH YOUNG AND MIDDLE-AGED ADULTS (15-69 years)

The International Physical Activity Questionnaires (IPAQ) comprises a set of 4 questionnaires. Long (5 activity domains asked independently) and short (4 generic items) versions for use by either telephone or self-administered methods are available. The purpose of the questionnaires is to provide common instruments that can be used to obtain internationally comparable data on health–related physical activity.

Background on IPAQ
The development of an international measure for physical activity commenced in Geneva in 1998 and was followed by extensive reliability and validity testing undertaken across 12 countries (14 sites) during 2000. The final results suggest that these measures have acceptable measurement properties for use in many settings and in different languages, and are suitable for national population-based prevalence studies of participation in physical activity.

Using IPAQ
Use of the IPAQ instruments for monitoring and research purposes is encouraged. It is recommended that no changes be made to the order or wording of the questions as this will affect the psychometric properties of the instruments.

Translation from English and Cultural Adaptation
Translation from English is supported to facilitate worldwide use of IPAQ. Information on the availability of IPAQ in different languages can be obtained at www.ipaq.ki.se. If a new translation is undertaken we highly recommend using the prescribed back translation methods available on the IPAQ website. If possible please consider making your translated version of IPAQ available to others by contributing it to the IPAQ website. Further details on translation and cultural adaptation can be downloaded from the website.

Further Developments of IPAQ
International collaboration on IPAQ is on-going and an ***International Physical Activity Prevalence Study*** is in progress. For further information see the IPAQ website.

More Information
More detailed information on the IPAQ process and the research methods used in the development of IPAQ instruments is available at www.ipaq.ki.se and Booth, M.L. (2000). *Assessment of Physical Activity: An International Perspective*. Research Quarterly for Exercise and Sport, 71 (2): s114-20. Other scientific publications and presentations on the use of IPAQ are summarized on the website.

We are interested in finding out about the kinds of physical activities that people do as part of their everyday lives. The questions will ask you about the time you spent being physically active in the **last 7 days**. Please answer each question even if you do not consider yourself to be an active person. Please think about the activities you do at work, as part of your house and yard work, to get from place to place, and in your sparetime for recreation, exercise or sport.

Think about all the **vigorous** activities that you did in the **last 7 days**. **Vigorous** physical activities refer to activities that take hard physical effort and make you breathemuch harder than normal. Think *only* about those physical activities that you did for at least 10 minutes at a time.

1. During the **last 7 days**, on how many days did you do **vigorous** physical activities like heavy lifting, digging, aerobics, or fast bicycling?

   _____ **days per week**

   ☐ No vigorous physical activities → ***Skip to question 3***

2. How much time did you usually spend doing **vigorous** physical activities on one of those days?

   _____ **hours per day**

   _____ **minutes per day**

   ☐ Don't know/Not sure

Think about all the **moderate** activities that you did in the **last 7 days**. **Moderate** activities refer to activities that take moderate physical effort and make you breathe somewhat harder than normal. Think only about those physical activities that you didfor at least 10 minutes at a time.

3. During the **last 7 days**, on how many days did you do **moderate** physical activities like carrying light loads, bicycling at a regular pace, or doubles tennis? Do not include walking.

   _____ **days per week**

   ☐ No moderate physical activities → ***Skip to question 5***

4. How much time did you usually spend doing **moderate** physical activities on oneof those days?

_____ **hours per day**

_____ **minutes per day**

☐ Don’t know/Not sure

Think about the time you spent **walking** in the **last 7 days**. This includes at work and at home, walking to travel from place to place, and any other walking that you have done solely for recreation, sport, exercise, or leisure.

5. During the **last 7 days**, on how many days did you **walk** for at least 10 minutesat a time?

_____ **days per week**

☐ No walking → ***Skip to question 7***

6. How much time did you usually spend **walking** on one of those days?

_____ **hours per day**

_____ **minutes per day**

☐ Don’t know/Not sure

The last question is about the time you spent **sitting** on weekdays during the **last 7 days**. Include time spent at work, at home, while doing course work and during leisure time. This may include time spent sitting at a desk, visiting friends, reading, or sitting orlying down to watch television.

7. During the **last 7 days**, how much time did you spend **sitting** on a **week day**?

_____ **hours per day**

_____ **minutes per day**

☐ Don’t know/Not sure

This is the end of the questionnaire, thank you for participating.

Table S1. The results of Kołmogorow-Smirnow's test and Brown-Forsythe's test for strategies of coping with stress.

Stage / Strategy	Stage I		Stage II		Stage III		Brown-Forsythe's test	
	d	p	d	p	d	p	F	p
**Active stress-coping methods**	0.21	**0.01**	0.21	**0.01**	0.22	**0.01**	0.73	0.482
**Planning**	0.21	**0.01**	0.20	**0.01**	0.22	**0.01**	6.44	**0.002**
**Positive revaluation**	0.19	**0.01**	0.15	**0.01**	0.17	**0.01**	11.73	**0.000**
**Acceptance**	0.22	**0.01**	0.17	**0.01**	0.16	**0.01**	35.04	**0.000**
**Sense of humour**	0.16	**0.01**	0.17	**0.01**	0.16	**0.01**	9.44	**0.000**
**Turning to religion**	0.25	**0.01**	0.25	**0.01**	0.24	**0.01**	2.90	0.055
**Seeking emotional support**	0.19	**0.01**	0.19	**0.01**	0.21	**0.01**	9.56	**0.000**
**Seeking instrumental support**	0.18	**0.01**	0.18	**0.01**	0.20	**0.01**	2.89	0.056
**Dealing with other things**	0.19	**0.01**	0.15	**0.01**	0.15	**0.01**	3.06	**0.047**
**Denial**	0.26	**0.01**	0.21	**0.01**	0.22	**0.01**	9.70	**0.000**
**Giving vent to one's feelings**	0.17	**0.01**	0.16	**0.01**	0.17	**0.01**	3.63	**0.027**
**Taking in psychopharmaceuticals**	0.43	**0.01**	0.40	**0.01**	0.38	**0.01**	20.73	**0.000**
**Doing nothing**	0.19	**0.01**	0.17	**0.01**	0.17	**0.01**	21.99	**0.000**
**Self-blaming**	0.16	**0.01**	0.14	**0.01**	0.11	**0.01**	5.27	**0.005**

Legend: d – "d score" in Kołmogorow-Smirnow's test; F – "F score" in Brown-Forsythe's test; p – significance level

Data were obtained with the Kołmogorow-Smirnow's test and Brown-Forsythe's test.

Table S2. The results of Kołmogorow-Smirnow's test and Brown-Forsythe's test for types of physical activity.

Stage	Stage I		Stage II		Stage III		Brown-Forsythe's test	
**Type of physical activity**	**d**	**p**	**d**	**p**	**d**	**p**	**F**	**p**
**Vigorous physical activity – Low**	0.28	**<0.01**	0.41	**<0.01**	0.35	**<0.01**	1.09	0.338
**Vigorous physical activity – Medium**	0.18	**<0.05**	0.24	**<0.01**	0.22	**<0.01**	0.75	0.473
**Vigorous physical activity – High**	0.14	<0.10	0.14	**<0.05**	0.17	**<0.05**	4.19	**0.16**
**Moderate physical activity – Low**	0.17	**<0.01**	0.39	**<0.01**	0.25	**<0.01**	0.97	0.378
**Moderate physical activity – Medium**	0.13	<0.10	0.29	**<0.01**	0.27	**<0.01**	1.79	0.169
**Moderate physical activity – High**	0.14	<0.15	0.18	**<0.05**	0.18	**<0.05**	7.67	**0.001**
**Walking – Low**	0.14	**<0.01**	0.24	**<0.01**	0.12	**<0.01**	3.93	**0.020**
**Walking – Medium**	0.15	**<0.05**	0.10	>0.20	0.17	**<0.05**	4.24	**0.015**
**Walking – High**	0.16	**<0.05**	0.15	**<0.05**	0.22	**<0.01**	0.97	0.382
**Sedentary time – Low**	0.16	**<0.01**	0.15	**<0.01**	0.29	**<0.01**	7.18	**0.001**
**Sedentary time – Medium**	0.26	**<0.01**	0.18	**<0.01**	0.32	**<0.01**	0.96	0.382
**Sedentary time – High**	0.16	**<0.05**	0.15	**<0.05**	0.20	**<0.01**	0.90	0.407

Legend: d – "d score" in Kołmogorow-Smirnow's test; F – "F score" in Brown-Forsythe's test; p – significance level

Data were obtained with the Kołmogorow-Smirnow's test and Brown-Forsythe's test.

Table S3. Association between coping with stress and progression of COVID-19 pandemic.

Strategy \ Stage	Stage II		Stage III		p[1]
	Median	X±SD	Median	X±SD	
**Active stress-coping methods**	2.00	2.15±0.76	2.00	2.08±0.81	0.127
**Planning**	2.00	2.04±0.80	2.00	2.01±0.83	0.921
**Positive re-evaluation**	1.50	1.43±0.86	1.50	1.47±0.91	0.121
**Acceptance**	2.00	1.72±0.80	2.00	1.67±0.82	0.479
**Sense of humour**	1.00	0.95±0.71	1.00	0.92±0.73	0.507
**Turning to religion**	0.50	0.92±1.00	0.50	0.95±1.02	-
**Seeking emotional support**	2.00	1.99±0.90	2.00	1.93±0.93	0.257
**Seeking instrumental support**	2.00	1.88±0.93	2.00	1.87±0.90	0.771
**Dealing with other things**	1.50	1.56±0.76	1.50	1.55±0.78	0.802
**Denial**	0.50	0.68±0.71	0.50	0.68±0.73	0.817
**Giving vent to one's feelings**	1.50	1.55±0.72	1.50	1.56±0.77	-
**Taking in psychopharmaceuticals**	0.00	0.37±0.65	0.00	0.44±0.71	0.114
**Doing nothing**	0.50	0.79±0.74	0.50	0.76±0.74	-
**Self-blaming**	2.00	1.70±0.93	1.50	1.67±0.95	0.732

Legend: X – average; SD – standard deviation; p – significance level; [1] – values obtained in the Dunn's test.

Data were obtained with the post-hoc Dunn's test.

Table S4. The correlations between stress-coping methods with age in the each examined stages of COVID-19 pandemic.

Correlations	Stage I		Stage II		Stage III	
	R	p	R	p	R	p
**Active stress-coping methods**	-0.033	0.228	-0.001	0.970	0.031	0.389
**Planning**	-0.014	0.605	0.099	**0.004**	0.021	0.570
**Positive reevaluation**	0.030	0.278	0.078	**0.025**	0.055	0.129
**Acceptance**	-0.015	0.586	0.067	0.053	0.022	0.538
**Sense of humour**	-0.020	0.474	-0.046	0.186	-0.030	0.406
**Turning to religion**	-0.027	0.334	0.048	0.164	0.019	0.600
**Seeking emotional support**	-0.006	0.825	0.025	0.478	0.038	0.300
**Seeking instrumental support**	-0.016	0.567	0.125	**0.000**	0.026	0.476
**Dealing with other things**	-0.050	0.072	0.084	**0.015**	0.077	**0.035**
**Denial**	0.024	0.388	-0.008	0.828	-0.000	0.995
**Giving vent to one's feelings**	-0.043	0.118	0.074	**0.033**	-0.021	0.568
**Taking in psychopharmaceuticals**	0.010	0.704	-0.026	0.459	0.042	0.246
**Doing nothing**	-0.034	0.219	0.019	0.581	-0.005	0.900
**Self-blaming**	-0.028	0.315	-0.025	0.479	-0.042	0.246

Legend: R – Spearman's rank correlation coefficient; p – significance level

Data were obtained with the Spearman's rank order test.

Table S5. Association between alcohol drinking pattern and progression of COVID-19 pandemic.

Stage / Number of points	Stage II N	Stage II %	Stage III N	Stage III %	χ2	OR	95% CI	p	Bonferroni correction
**<8 points**	554	78.58%	470	68.71%	10.66	1.67	1.23-2.28	**0.001**	0.0042
**8-15 points**	133	18.87%	189	27.63%	32.01	2.80	1.95-4.02	**<0.000**	0.0042
**16-19 points**	8	1.13%	11	1.61%	4.47	2.71	1.04-7.04	0.346	0.0042
**>20 points**	10	1.42%	14	2.05%	5.69	2.76	1.17-6.53	0.017	0.0042

Legend: χ2 – "χ2 score" in chi square test; OR – odds ratio; 95% CI – confidence interval; p – significance level

Data were obtained with the chi square test.

Table S6. Association between smoking pattern and progression of COVID-19 pandemic.

Stage / Number of points	Stage II N	Stage II %	Stage III N	Stage III %	χ2	OR	95% CI	p	Bonferroni correction
**0-4 points**	54	88.52%	93	94.90%	16.46	0.49	0.34-0.69	**0.000**	0.0056
**5-8 points**	6	9.84%	4	4.08%	0.0015	1.26	0.35-4.49	0.969	0.0056
**9-11 points**	1	1.64%	1	1.02%	0.3446	0.84	0.05-13.46	0.557	0.0056

Legend: χ2 – "χ2 score" in chi square test; OR – odds ratio; 95% CI – confidence interval; p – significance level

Data were obtained with the chi square test.

Table S7. Association between physical activity and progression of COVID-19 pandemic.

Stage / The intensity of effort	Stage II		Stage III		χ2	OR	95% CI	p	Bonferroni correction
	N	%	N	%					
**Low**	631	74.68	580	76.82	1.00	0.89	0.71-1.12	0.318	0.0056
**Medium**	108	12.78	90	11.92	0.87	0.87	0.64-1.17	0.351	0.0056
**High**	106	12.54	85	11.26	0.14	0.94	0.70-1.27	0.704	0.0056

Legend: χ2 – "χ2 score" in chi square test; OR – odds ratio; 95% CI – confidence interval; p – significance level

Data were obtained with the chi square test.

Table S8. Association between physical activity (broken down by exercise intensity (Low, Medium, High) and type of physical activity) and progression of COVID-19 pandemic.

"Low", "Medium" and "High" together			
**Vigorous physical activity [MET] – Median**	**Moderate physical activity [MET] – Median**	**Walking [MET] – Median**	**Sedentary time [MET] – Median**
Stage II – 320, Stage III – 160 **p=0.005**	Stage II – 240, Stage III – 160 **p=0.011**	Stage II – 693, Stage III – 792 **p=0.001**	Stage II – 300, Stage III – 240 **p<0.0001**

**Low**		**p**	**Medium**		**p**	**High**		**p**
**Vigorous physical activity [MET] – Median**			**Vigorous physical activity [MET] – Median**			**Vigorous physical activity [MET] – Median**		
**Stage II**	**Stage III**		**Stage II**	**Stage III**		**Stage II**	**Stage III**	
420	480	**0.030**	960	720	-	2160	1920	-
**Low**		**p**	**Medium**		**p**	**High**		**p**
**Moderate physical activity [MET] – Median**			**Moderate physical activity [MET] – Median**			**Moderate physical activity [MET] – Median**		
**Stage II**	**Stage III**		**Stage II**	**Stage III**		**Stage II**	**Stage III**	
160	90	-	280	240	0.527	480	680	-
**Low**		**p**	**Medium**		**p**	**High**		**p**
**Walking [MET] – Median**			**Walking [MET] – Median**			**Walking [MET] – Median**		
**Stage II**	**Stage III**		**Stage II**	**Stage III**		**Stage II**	**Stage III**	
676.5	693	**0.005**	693	792	0.103	924	1386	-
**Low**		**p**	**Medium**		**p**	**High**		**p**
**Sedentary time [MET] – Median**			**Sedentary time [MET] – Median**			**Sedentary time [MET] – Median**		
**Stage II**	**Stage III**		**Stage II**	**Stage III**		**Stage II**	**Stage III**	
300	240	**<0.0001**	240	300	**0.001**	300	300	0.055

Legend: p – significance level

Data were obtained with the post-hoc Dunn’s test.
